# 

**Published:** 1861-11

**Authors:** 


### CONTENTS.---NOVEMBER, 1861.

### ORIGINAL CONTRIBUTIONS.

PAGE

ART. I. Lectures on Military Surgery. By E. ANDREWS, A. M., M. D., Professor of Surgery, etc. Lecture VII, .......... 577

ART. II. Fragilitas Ossium. By ORRIN SMITH, A. M., M. D., Chicago, Ill., .......... 584

ART. III. The Surgeon. An Introductory Lecture, Delivered before the Med. Dept. Lind University, Session 1861-62. October 14, 1861. By Prof. E. ANDREWS, M. D., 587

ART. IV. The Mercurial Treatment of Morbus Coxæ. By W. GODFREY DYAS, F.R.C.S., 598

### THE CLINIQUE.

CHICAGO MEDICAL AND SURGICAL DISPENSARY.—Service, Prof. E. ANDREWS, M. D., Attending Surgeon. Gun-Shot Wounds—Fracture of Bones of Forearm. by Minie Bullet, .......... 607

Service, Prof. WM. H. BYFORD, M. D., Attending Physician. Ozœna, .......... 610

Additional Cases, from a paper on the "Use of Inhalations in the Treatment of Inflammations of the Nasal Passages and their Appendages." By FRANK W. REILLY, M. D. 613

### SELECTIONS.

The Memoirs of Dr. MARSHALL HALL, .......... 616

Amputation of the Cervix Uteri. By J. MARION SIMS, M. D., Surgeon to the Woman's Hospital, New York, .......... 621

ITEMS: Dilute Glycerine, 583. Suggestions to Soldiers, .......... 586

### BOOK NOTICES.

Report of a Committee of the Boston Society, for Medical Improvement, on the Alleged Dangers which Accompany the Inhalation of the Vapor of Sulphuric Ether, .......... 625

THE PATHOLOGY AND TREATMENT OF VENEREAL DISEASES including the Results of Recent Investigations on the Subject. By FREEMAN J. BUMSTEAD, M. D., Lecturer on Venereal Diseases, at the College of Physicians and Surgeons, New York; Surgeon to St. Luke's Hospital; Assistant-Surgeon to the New York Eye Infirmary, etc. .......... 634

### EDITORIAL.

Medical Department of Lind University .......... 638

Local Medical Societies .......... 639

Communications Received .......... 639

Close of the Volume .......... 639

Rush Medical College .......... 639

Scurvy in the Missouri Army .......... 637

